# FEAR OF CANCER RECURRENCE IN OLDER WOMEN IN REMISSION FROM GYNAECOLOGICAL CANCER IN REGIONAL AUSTRALIA

**DOI:** 10.1093/geroni/igad104.3349

**Published:** 2023-12-21

**Authors:** Senara Kulatunga, Stacey Rich, Christopher Steer, Tshepo Rasekaba, Nicole Webb, Irene Darmadi Blackberry

**Affiliations:** University of New South Wales, Albury, New South Wales, Australia; La Trobe University, Wodonga, Victoria, Australia; Border Medical Oncology, Albury, New South Wales, Australia; La Trobe University, Melbourne, Victoria, Australia; Albury Wodonga Health, Albury, New South Wales, Australia; La Trobe University, Wodonga, Victoria, Australia

## Abstract

Fear of cancer recurrence (FCR) can be a constant feature of cancer survivorship and can negatively impact quality of life. Whilst the symptom burden from FCR is higher in younger patients, the impact of FCR in older adults is poorly understood. This project explores FCR, survivorship and the coping strategies associated with FCR in older women in remission after treatment of gynaecological cancer, a cancer type with high recurrence rates. Qualitative interviews were conducted with ten older women (age range 68-87 years) in remission from gynaecological cancer (Ovarian = 5, Uterine = 5). Interviews were designed to explore the experience of FCR in older women who had been treated at a regional cancer centre in Australia. Thematic analysis was undertaken to uncover themes related to the experience of FCR and coping strategies employed. Emergent themes included fear of burdening others, the emotional impact of the death of close others, and positive or negative experience of prior treatment. Emergent themes in coping with FCR in survivorship included helping others, comparison to others, keeping occupied, and support from others. Patients discussed preferring not to dwell on thoughts of recurrence. These findings are novel in FCR literature as they point to an experience of FCR in older women that may not fit the profile of the general population. Whilst further work is needed, these findings support the need for a tailored approach to supporting older women through survivorship to ensure optimal quality of life.

